# Discovering healthcare provider behavior patterns through the lens of Medicare excess charge

**DOI:** 10.1186/s12913-020-05876-1

**Published:** 2021-01-04

**Authors:** Sagnika Sen, Amit V. Deokar

**Affiliations:** 1 School of Graduate Professional Studies, Pennsylvania State University, Malvern, PA 19355 USA; 2 Robert J. Manning School of Business, University of Massachusetts Lowell, Lowell, MA 01854 USA

**Keywords:** Reimbursement policy, Payment variation, Medicare, Two-step cluster analysis, Healthcare provider behavior

## Abstract

**Background:**

The phenomenon of excess charge, where a healthcare service provider bills Medicare beyond the limit allowed for a medical procedure, is quite common in the United States public healthcare system. For example, in 2014, healthcare providers charged an average of 3.27 times (and up to 528 times) the allowable limit for cataract surgery. Previous research contends that such excess charges may be indicative of the actual amount that providers bill to non-Medicare patients and subsequent cost-shifting behavior, where a healthcare provider tries to recoup underpayment by Medicare from privately insured, self-pay, out-of-network, and uninsured patients.

**Objectives:**

The objective of this study is to examine the drivers of a provider’s excess charge patterns, especially the extent to which the degree of excess charges may be associated with physician characteristics, Medicare reimbursement policy, or socioeconomic status and demographics of a provider’s patient base.

**Methods:**

Using data from the 2014 Medicare Provider Utilization files, we identify three procedures with the highest variation in Medicare reimbursements to study the excess charge phenomenon. We then employ a two-step cluster analysis within each procedure to identify distinct provider groups.

**Results:**

Each procedure code yielded distinct healthcare provider segments with specific patient demographics and related behavior patterns. Cluster silhouette coefficients indicate that these segments are unique. Three random subsamples from each procedure establish the stability of the clusters.

**Conclusions:**

For each of the three procedures investigated in this study, a sizeable number of healthcare providers serving poorer, riskier patients are often paid significantly lower than their peers, and subsequently have the highest excess charges. For some providers, excess charges reveal possible cost-shifting to private insurance. Patterns of excess charges also indicate an imbalance of market power, especially in areas with lower provider competition and access to health care, thus leading to urban-rural healthcare disparities. Our results reinforce the call for price transparency and an upper limit to overbilling.

**Supplementary Information:**

The online version contains supplementary material available at 10.1186/s12913-020-05876-1.

## Background

The phenomenon of excess charge, where a healthcare service provider bills Medicare (the federal government insurance program for people 65 and older) beyond the limit allowed for a medical procedure or treatment, is quite well known in the United States public healthcare system [1–3]. Medicare and Medicaid (joint state and federal programs for low-income families or individuals) respectively covered 13 and 19% of the US population in 2014; 55% were covered by private insurance [4]. Reimbursement by Centers for Medicare and Medicaid Services (CMS, the administrator of both Medicare and Medicaid) to a healthcare service provider for a specific medical service for Medicare beneficiaries follows a formula adjusted for differences in cost-of-living expenses from one geographical region to another [5]. Given these adjustments, some variation in provider billing is expected. Interestingly, despite the formula-based reimbursements by Medicare, the variation in average provider billing amounts for a specific medical service is beyond what can be explained by geography alone [6]. Given that healthcare providers cannot get reimbursed beyond the amount that Medicare deems reasonable, this has led to a vigorous debate. It has been argued in the literature [1, 3] that excess charges may be indicative of consistently high prices charged by a provider to non-Medicare patients [1, 3]. Evidently, the most adverse effects of such overpricing are experienced by the uninsured and accidental out-of-network patients, as they are forced to pay the full price [7–9]. Private insurance data are not always readily available. However, when such data are accessible, studies have shown that private and public insurance payment patterns in the US have often followed opposite trends within a given region [10]. In other words, persistent overbilling by certain provider segments may be indicative of underpayment by Medicare, a phenomenon that has been observed for some healthcare services [11, 12] and is known as cost-shifting, where providers increase the price for private insurance to account for the shortfall from Medicare [13, 14].

Excess charge, also referred to as markup ratio, has been explored in the literature from the perspective of physician specialty and region [1], hospital characteristics (e.g., for-profit vs. nonprofit) [15], emergency department and internal medicine [2], and critical care [3]. While these works have made significant contributions toward revealing patterns of excess charge behavior, more research is called for to include patient characteristics and practice patterns that may also affect physician charges [3]. In this regard, our objective is to investigate how factors related to the medical procedure, provider’s practice, and demographic and socioeconomic factors of the patient base are associated with excess charge. We specifically focus on procedures with high payment (i.e., reimbursement) variation. Payment variation refers to the wide range of reimbursements to clinicians by health insurance (both private and public) for similar services [16–20]. These variations are substantial, even after adjusting for geographic and cost-of-living differences [20, 21].

Given that Medicare allowed and payment (reimbursement) amounts show a very high degree of positive correlation, our objective is to explore whether excess charge (the ratio of submitted charges to the allowed amount for a medical service) is linked with payment variation in any way; more importantly, what does excess charge inform us regarding provider behavior patterns for medical services with high payment variation? Both excess charge and payment variation have been cited in the literature as possible indicators of healthcare disparity and quality issues [18, 22]. Previous research has primarily focused on measuring the extent of variation within a particular specialty/discipline such as radiation oncology [23] or surgery [24]. In this research, we use high payment variation as the selection criterion to further our understanding of the source and cause of a provider’s excess charge behavior. To the best of our knowledge, the association of a specific medical service, healthcare provider characteristics, and patient demographics with excess charge and payment variation has not yet been studied. Because healthcare spending in the US is almost double that of other high-income countries, with the cost of medical labor being one of the major contributing factors for such a difference [25], we argue that it is essential to explore these associations.

In this research, we explore healthcare providers’ behavior patterns with respect to the amount billed (price) to Medicare by focusing on four dimensions. *Healthcare Provider* captures information about a specific medical service provider such as gender, credentials, and affiliation. Attributes related to *Medical Procedure* capture information regarding a specific medical service (e.g., screening mammogram) such as volume, the number of unique beneficiaries served, place of service, payment received, etc. Attributes related to *Medical Practice* capture information regarding the entire gamut of services provided by the healthcare provider. These include aggregate information about all services offered by a provider, such as the number and volume of different medical procedures performed, and the total payment received from Medicare. In addition, demographic information such as age, risk score, race, gender distribution, and socioeconomic information such as health risk and poverty (with respect to the medical practice’s patient pool) are captured under *Demographic and Socioeconomic* attributes.

In 2014, 37 medical procedures accounted for almost half ($40.64B) of Medicare Part B Fee-For-Service payments of $78.22 billion, covering 5973 unique procedure codes. We focus on the three procedures within these top 37 that exhibit the highest degree of payment variation. These procedures, as identified by the Healthcare Common Procedure Coding Systems (HCPCS), are – 66,984: non-complex cataract surgery, G0202: screening mammograms, and 78,452: nuclear imaging and study of cardiac health.

For each of these three medical procedures, we employ a two-step cluster analysis technique to investigate the healthcare provider, medical procedure, medical practice, and demographic and socioeconomic attributes to identify provider segments in the context of excess charge. Cluster analysis is a novel approach used to investigate healthcare price variation. Prior research in this stream has mostly pre-categorized providers into high-vs-low price groups [2, 15, 22]. A segmentation strategy allows pricing behavior to be explored in conjunction with other relevant factors. Subsequently, our analysis offers unique insights regarding how the complex interplay of CMS payment policy and patient demographics may lead to specific billing patterns by different groups of providers. Our work thus contributes to the literature on healthcare price and payment variation by adopting a holistic approach to identify systematic behavior patterns, and more importantly, potential factors leading to such behavior.

In the following, we discuss the relevant literature in price variation and the rationale for using cluster analysis. Definitions of the key terms used in this paper are provided in Table 1.

**Table 1:** Definitions of Key Terms

Term	Definition
Medical Procedure or Service	Any medical service or doctor visit, coded as per the Healthcare Common Procedure Coding System (HCPCS).
Healthcare Provider	Physicians, laboratories, and clinics providing diagnostic services, etc.
Medical Practice	The entire gamut of medical services provided by the healthcare provider.
Price/Charge	The amount a provider bills (for a specific service) to the insurance or to an uninsured or out-of-network patient.
Payment	The amount a healthcare provider received (i.e., reimbursement) for a medical procedure. For insured patients, the insurer pays a pre-negotiated amount. Uninsured or out-of-network patients often pay the full price.
Allowable Limit	The maximum total amount for a specific service, including the insurance payment, co-pay, and/or coinsurance, and any other third-party payments, if applicable.
Premium	Monthly fee paid to the health insurer in order to be covered by a specific health insurance plan.
Deductible	(optional) Yearly amount to be paid by the insured before the health insurance plan covers the medical bills.
Copay	A predetermined amount/rate paid to a healthcare provider at the time of care may vary by provider specialty.
Coinsurance	(optional) A percentage of medical charges paid by the insured; the rest is paid by the health insurance plan. A 20% coinsurance means that 20% of each medical bill is paid by the insured, and the health insurance covers 80%.
Out of pocket	The total amount the insured pays. This includes the copay, coinsurance, and deductibles, if any. Federal rules limit the maximum out-of-pocket expenses for insured patients for every plan year.
Network/in-Network	Individual or group of healthcare providers recognized by the health insurance plan. Private health insurers negotiate confidential payment terms (i.e., prices for each service rendered) with these providers, who agree to accept patients covered by the insurer.
Out of network	Individual or group of healthcare providers who do not have a negotiated a price contract with the health insurer. In case the insured gets care from an out-of-network provider, they may have to pay the entire medical bill, or a portion thereof, as indicated in their healthcare plan.

### Price and payment variation in medicare

Price variation in various medical procedures and tests across the US is well known and spans across the spectrum of medical services [26]. There are abundant examples in both private and public insurance. Within Medicare, considerable variation in payment patterns for surgery is driven by large-scale office-based events and by a disproportionate number of services by few physicians [27]. In radiation oncology, a significant amount of variation arises from technical vs professional fees. Gender and rurality also contribute to the variation [28]. High variation in episode-based payments involving urologic cancer surgery [29] and coronary artery bypass grafting [30] made a call for bundled payments instead of the fee-for-service payment structure in most Medicare plans. Variations are also observed in commercial insurance programs [31, 32]. Private insurer payments for routine physician office visits vary significantly [17]. While some of the price variation is explained by geography, a great deal of unexplained variation still remains.

In search of the drivers of variation, studies have focused on excess charge [2] or markup [15], the ratio of submitted charges to the total allowable limit for a service. As with payments, wide variation in the markup ratio is observed nationwide. For hospitals in the US, excess charge is shown to be a function of state-level price-setting policies, as well as the for/not-for profit status of hospitals [15]. It is also shown that hospital excess charges for the emergency department (ED) may vary substantially, and hospitals charge more when patients are seen by an ED physician compared to an internal medicine physician [2]. Our work adds to this literature by focusing on provider behavior with respect to specific procedures and by linking Medicare payment with excess charge.

### Provider behavior patterns and cluster analysis

Driven by the escalating costs of healthcare in the US, policymakers, insurance companies, and managed care providers have engaged in creating profiles of healthcare providers such as physicians, hospitals, and clinical facilities by linking cost and efficiency [33–36]. Our current work complements this stream of research in that our aim is to reveal the associations, if any, between specific medical procedures and a healthcare provider’s practice (the current literature focuses on either of them, but not both), and their relationship to patient demographics. In this respect, cluster analysis, with its roots in market segmentation, provides an innovative approach to segment the data into a set of homogeneous and meaningful natural groupings by identifying latent structures in the data [37]. While this method has been used in other areas of healthcare, e.g., classifying patient populations with different morbidity patterns [38, 39], identifying patterns of white matter abnormalities in patients with schizophrenia [40], and identifying physician-patient interaction styles [39], it has not been used to explore *whether* and *how* a provider, practice, and patient characteristics interact in unique ways.

Cluster analysis is a statistical technique that finds applications in many areas due to its ability to summarize and understand vast amounts of data. Observations that are similar (or related) to each other based on various characteristics are grouped together, while those that are different (or unrelated) are not. Thus, clusters are subgroups of observations whose differences are traditionally measured by some sort of distance measure (e.g., Euclidean distance). Clustering algorithms operate iteratively to determine clusters of observations in which the observations within each cluster are similar to one another, i.e., homogeneous, while the clusters themselves are distinct from one another.

Among the three key types of cluster analysis techniques, namely k-means clustering, hierarchical clustering, and two-step clustering, we employ two-step clustering in this study for the following reasons [37]. K-means clustering assumes a specific number of *k* clusters prior to analysis, and while a different analysis may be conducted based on different values of *k*, there is no strong rationale for this strategy. Hierarchical clustering is commonly used to generate a series of cluster solutions ranging from the least to most granular and works best when the variables are of a similar type (e.g., numeric scaled data). Both k-means and hierarchical clustering algorithms are typically designed to work on continuous variables, whereas the two-step algorithm handles both categorical and continuous variables. As such, it does not require any data transformation and retains full information, which is helpful in interpreting the practical implications of the clusters emerging from the analysis [41]. Lastly, the algorithm can be scaled to large datasets such as the one in this study [42]. Given the suitability of this approach to the study objectives, two-step cluster analysis using IBM SPSS software [43] was performed.

## Method

### Two-step cluster analysis

The objective of our study is to take an exploratory stance in terms of analyzing the collective effect of all relevant attributes in finding out how the observations would relate to or separate from one another. We set out to understand the distribution of the data without a priori hypotheses. Hence, we opted to use cluster analysis as the methodology in this study.

The two-step cluster analysis consists of two stages: pre-clustering and clustering [44]. In the first stage, the observations are grouped into so-called *pre-clusters* by building a clustering feature tree (CF-tree) with a sequential scan of the data. The pre-clustering is based on the BIRCH (*Balanced Iterative Reducing and Clustering using Hierarchies*) approach originally proposed by Zhang et al. [45] for continuous attributes, and later refined by Chiu et al. [44] to handle both continuous and categorical attributes. The CF-tree data structure transforms individual observations into cluster features that contain essential summary characteristics of a dense region of observations. Using Chiu et al.’s [44] notation, the cluster feature *CF*_*j*_ of a cluster *C*_*j*_ is: ${CF}_j=\left\{{N}_j,{s}_{Aj},{s}_{Aj}^2,{N}_{Bj}\right\}$, where *N*_*j*_ is the number of data records in *C*_*j*_, *s*_*Aj*_ is the sum of continuous attributes of *N*_*j*_ data records, ${s}_{Aj}^2$ is the sum of the squared continuous attributes of *N*_*j*_ data records, and *N*_*Bj*_ is a vector of dummy-coded categorical attributes.

In the second stage, the pre-clusters are used to first determine the number of clusters automatically, and then each observation is assigned to a cluster. In determining the number of clusters, first a rough estimate is generated based on the Bayesian information criterion (BIC), which is a likelihood criterion that penalizes model complexity, as determined by the number of parameters in the model. In other words, models with lower BIC values are preferred models. As the number of clusters increases, the BIC value starts to decrease before increasing again. This model-fitting behavior helps automatically determine the number of clusters by analyzing when BIC has the lowest value [44]. Following this, the algorithm assumes independence among the variables and normal and multinomial distributions for each numerical and categorical variable, respectively. A joint normal-multinormal distribution is used to find the log-likelihood of each case to belong to a specific cluster.

### Cluster validation

#### Silhouette measure

A good quality cluster would have high intra-class similarity, i.e., cluster cohesion and low interclass similarity, i.e., cluster separation. A popular metric called the Silhouette Coefficient [46] combines both cohesion and separation to produce an overall measure of cluster validity. The Silhouette Coefficient ranges from − 1 to 1 and is the average silhouette value of all observations in the data. A higher coefficient indicates that on average, each observation is well matched (high similarity, indicating cohesion) to its designated cluster and poorly matched (high dissimilarity, indicating separation) with other clusters. In this study, we adopt this metric to assess cluster quality.

#### Cluster stability

Further, clusters should be demonstrated as being stable, i.e., they should not be the result of the idiosyncrasies of a particular dataset; rather, they should represent a consistent pattern. An established method of ensuring such stability is to apply the same clustering algorithm to random subsamples [38, 40, 47]. The subsampling strategy aims to determine if perturbations in the data will present different cluster analysis results.

## Application

### Data

We used the 2014 Provider Utilization and Payment Data: Physician and Other Supplier from Medicare. In 2014, total Medicare payments for Part B Fee-For-Service amounted to $78.22 billion, covering 5973 unique medical procedure codes. Of these, 37 HCPCS accounted for almost half ($40.64B) of Medicare’s total spending. Within these most expensive procedures, three of them show abnormally high coefficients of variation (CoV = standard deviation/mean) in terms of payment per service. Given that the average price for a procedure varies widely, depending on the complexity and resource requirements, CoV is a useful measure to gauge the degree of variability within each procedure. The coefficients of variation for procedure codes 66,984, G0202, and 78,452 are 0.65, 0.84, and 0.63, respectively, while the same for the remaining 34 services range from 0.01 to 0.20, thereby indicating a much higher degree of variability as to how providers are reimbursed.

For each procedure code (identified by HCPCS), we combine two files. The first file contains detailed information about the medical procedure such as the service volume, # of unique beneficiaries, and average payment and charge amounts serviced by a healthcare provider. A second file contains healthcare providers’ aggregate payment and charge amounts, along with the providers’ medical practice-level information such as the geographic location, beneficiary risk score, and racial and gender breakdown. These two files are combined using the provider’s National Provider Identification (NPI). A separate file from CMS with Urban/Rural/Very Rural indicators by 5-digit zip codes was combined with this data set.

### Attributes

The variables included in our cluster analysis is provided in Table 2. In addition, we calculated the following ratios to derive behavior patterns:

**Table 2:** Attributes used in the analysis

Attribute	Example/Definition
*Healthcare Provider*	
Type (Specialty)	Cardiology, Family Practice, …
Gender	Male/Female
Credential	M.D.; D.O.; CRNP,…
Entity Code	Individual/Organization
*Medical Procedure*	
Place of Service	Office/Facility
Service Volume	Number of services performed
Number of Unique Beneficiaries	Number of distinct Medicare beneficiaries receiving the service
Number of Visits	Number of distinct Medicare beneficiary/per day services
Number of Services Per Unique Beneficiary^a^	Number of Services per unique beneficiary
Number of Service Per Visit^a^	Number of services per visit
Medicare Allowed Amount	Average of the Medicare allowed amount for the service
Submitted Charge Amount	Average of the charges that the provider submitted for the service
Medicare Payment Amount	Average amount that Medicare paid
Excess Charge Ratio^a^	Average Submitted Charge Amount/ Average Medicare Allowed Amount
*Medical Practice*	
Total Number of Unique Procedures	Total number of unique services provided by the provider
Total Service Volume	Total services performed by the provider across all procedures
Total Number of Unique Beneficiaries	Total number of distinct Medicare beneficiaries serviced by the provider
Total Medicare Allowed Amount	Total Medicare allowed amount for all services
Total Submitted Charge Amount	Total charge the provider submitted for all services
Total Medicare Payment Amount	Total amount that Medicare paid to the provider
Overall Excess Charge Ratio^a^	Total Submitted Charge amount/ Total Medicare Allowed amount
*Demographic and Socioeconomic*	
Location	Urban/Rural/Very Rural
Beneficiary Average Age	% of the provider’s beneficiaries qualifying for both Medicare and Medicaid
Beneficiary Male %^a^	% of male beneficiaries
Beneficiary Female %^a^	% of female beneficiaries
Beneficiary White %^a^	% of beneficiaries who identify themselves as non-Hispanic white
Beneficiary Average Risk Score	Average age of the beneficiaries serviced by the provider
Beneficiary Dual Percentage^a^	Average risk score of beneficiaries serviced by the provider

attributes marked with ^a^ are derived attributes

#### Service per unique beneficiary [27]

This reflects the average number of times the same medical procedure is performed per patient. While the total service volume may vary widely from provider to provider, a relatively higher value of this ratio may be indicative of systematic overtreatment.

#### Service per visit

This is the number of medical procedures performed per patient for every unique patient-provider encounter. Relatively higher values of this ratio may be a cause for concern as well.

#### Excess charge ratio

This is the ratio of charges that the provider bills for a particular medical procedure to the Medicare allowable amount. This ratio has also been referred to as excess charge [1] or the markup ratio [2]. An excess charge ratio of 3 would mean that for a $100 Medicare allowable amount, the provider billed $300 on average. That is, the $200 excess charge can be assumed to be billed to uninsured and out-of-network patients in the absence of a provider discount [1, 2].

#### Overall excess charge ratio

This is the ratio of the total amount that the healthcare provider bills to Medicare for all of its services to the total amount Medicare pays. It is similar to the medical procedure level excess charge ratio but provides information about a provider’s medical practice and the billing patterns of the practice as a whole.

In addition, the beneficiary dual, beneficiary male/female, and beneficiary female percentages are calculated by dividing the number of beneficiaries in each category by the total number of unique beneficiaries serviced by the provider. Using percentages, as opposed to absolute counts for these demographic variables, is used as a standardization measure. Of these variables, the beneficiary dual percentage reveals important information about the practice economy, given that people with dual eligibility are usually from lower income strata and in need of long-term care [48].

## Results

Results of the two-step cluster analysis on the three procedure codes are provided here. Procedure code 66984 resulted in three distinct provider clusters, while procedure codes 78,452 and G0202 resulted in four distinct clusters. Distinct provider behavior patterns revealed in each cluster are discussed next.

### HCPCS 66984: removal of cataract with the insertion of lens

Of the three medical procedures chosen, this one accounts for the most Medicare spending. The results of the two-step cluster analysis are provided in Table 3, highlighting the variables with the most significant differences among the clusters. The cluster silhouette measure of cohesion and separation is 0.5 (considered fair). The clusters predominantly align along a combination of provider type and place of service. The largest provider cluster, #3, is composed of ophthalmologist facility visits, while the smallest provider cluster (#2) represents ambulatory surgical centers (ASC). Optometrist office visits are represented in cluster #1.

**Table 3:** Healthcare Provider Clusters: HCPCS Code 66984

		Cluster Centroids (Average)		
	Cluster # Relative Size (Frequency)	1 31% (5403)	2 11% (1867)	3 58% (9908)
**Healthcare Provider Attributes**	Provider Type	Optometry (99.29%)	Ambulatory Surgical Center (99.8%)	Ophthalmology (99.8%)
**Medical Procedure Attributes**	Place of Service	Office (94.9%)	Facility (99.9%)	Facility (97.9%)
	Service Volume	649.43	623.32	223.55
	Unique Beneficiary	25.27	404.33	110.27
	Service/Unique Beneficiary	27.76	1.51	2.38
	Medicare Allowed Amount	$94.15	$961.84	$612.69
	Medicare Payment Amount	$73.37	$745.52	$474.28
	Excess Charge Ratio	2.32	4.30	3.60
**Medical Practice Attributes**	Total Unique HCPCS codes submitted	18.01	92.86	39.07
	Total Unique Beneficiary	381.68	974.02	953.69
	Overall Excess Charge Ratio	1.31	4.34	2.28
**Demographic and Socio-economic Attributes**	Beneficiary Dual Percentage	18.77	15.40	16.89
	Location Mix (Urban: Rural: Very Rural)	(48%: 40%: 12%)	(84%: 13%: 3%)	(86%: 12%: 2%)

Cluster #1, optometrists, have a higher number of services per visit and services per unique beneficiary. Due to the nature of the service being postoperative care, the payment amount is substantially lower compared to the other two provider clusters.

**Fig. 1:**
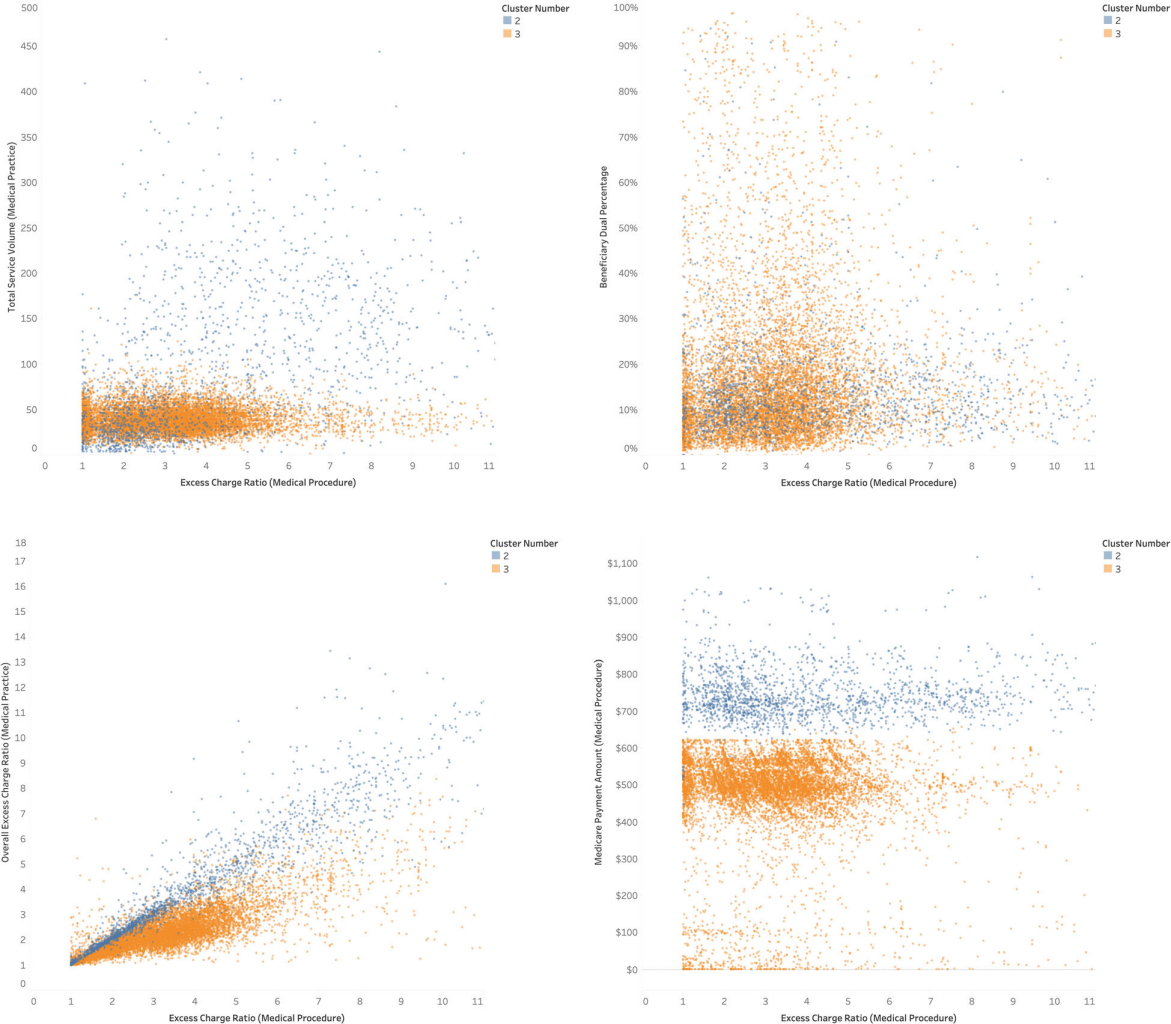
Cluster Differences for Cataract Surgery (Medical Procedure 66984)

The most salient difference, though, is between ophthalmologists (cluster #2) and ASCs (cluster #3). For the same cataract surgery, an average ASC charges 4.30 times the Medicare allowed amount and is paid $745 compared to an ophthalmologist, who charges by a factor of 3.60 and is paid $474. In 2014, the average ASC serviced 623 such surgeries compared to 223 for an ophthalmology facility. At the practice level too, ASCs charge 4.34 times compared to 2.28 times for ophthalmology facilities. A scatter plot of selected attributes for clusters #2 and #3 are illustrated in Fig. 1.

Another noticeable aspect is the relative geographic distribution of the providers. Cluster #1, which is primarily composed of optometrists, has the highest relative distribution in rural and very rural areas, 48 and 12%, respectively. ASCs and ophthalmology offices, on the other hand, have a much smaller presence in such areas.

### HCPCS 78452: nuclear medicine study of vessels of the heart using drugs or exercise, multiple studies

Of the three medical procedures chosen, cardiovascular imaging services have the highest degree of payment variation. A two-step cluster analysis of the data yielded 4 distinct clusters, with a cluster silhouette measure of cohesion and separation of 0.3 (marked fair). Table 4 highlights the variables with the most significant differences among the clusters.

**Table 4:** Healthcare Provider Clusters: HCPCS Code 78452

		Cluster Centroids (Average)			
	Cluster # Relative Size (Frequency)	1 35% (7153)	2 39.3% (7882)	3 20.0% (4006)	4 5% (993)
**Healthcare Provider Attributes**	Provider Type	Cardiology (83.8%)	Cardiology (89.6%)	Diagnostic Radiology (97.4%)	Cardiology (72.5%)
**Medical Procedure Attributes**	Place of Service	Office (100%)	Facility (94.9%)	Facility (97.7%)	Office (82.2%)
	Service Volume	108.39	96.41	53.74	324.18
	Unique Beneficiary	86.703	84.846	51.169	279.283
	Service/Unique Beneficiary	1.01	1.01	1.00	1.18
	Medicare Allowed Amount	$473.02	$80.09	$81.87	$343.88
	Medicare Payment Amount	$364.42	$60.35	$61.16	$264.49
	Excess Charge Ratio	2.59	4.04	3.87	3.97
**Medical Practice Attributes**	Total Unique HCPCS codes submitted	72.53	61.45	178.94	104.63
	Overall Excess Charge Ratio	0.86	1.18	1.30	2.45
**Demographic and Socio-economic Attributes**	Beneficiary Dual Percentage	22.19	23.06	28.82	25.73
	Location Mix (Urban: Rural: Very Rural)	(92%: 8%: 0%)	(87%: 12%: 1%)	(82%: 15%: 3%)	(82%: 17%: 1%)

The clusters are formed primarily according to the combination of healthcare provider type and place of service. Provider clusters #1, #2, and #3 represent cardiology offices, cardiology facilities, and diagnostic radiology facilities, respectively, while cluster #4 represents a small subset of cardiology offices, with high excess charges both at the medical procedure and medical practice levels.

**Fig. 2:**
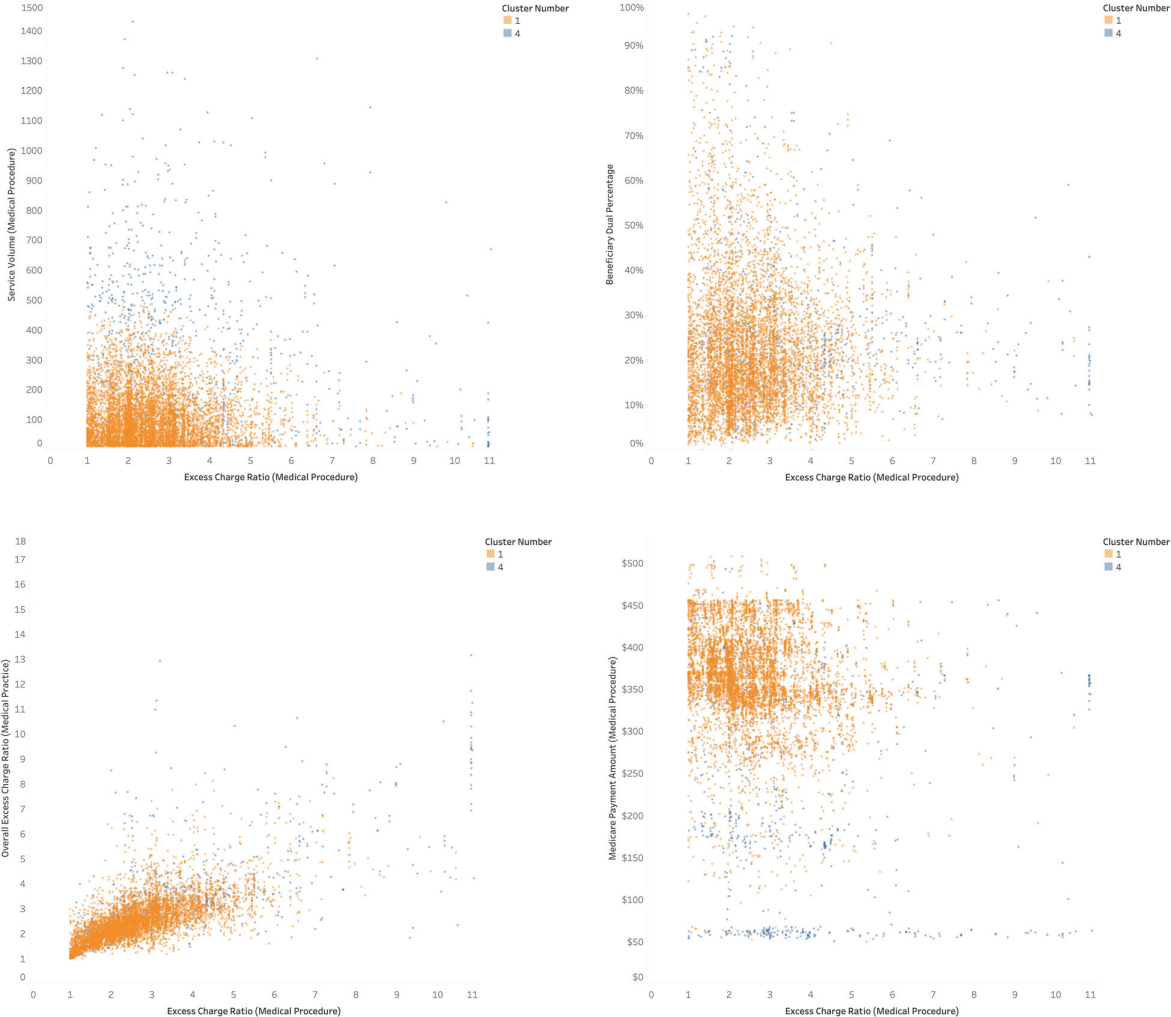
Cluster Differences for Cardiovascular Imaging Services (Medical Procedure 78,452)

The most remarkable differences are between clusters #1 and #4. While both clusters represent cardiology offices, cluster #4 (which consists of 5% of all providers), is characterized by the highest excess charge ratio at the medical practice level (2.45), and second highest excess charge ratio (3.97) at the medical procedure level. Cluster #1, cardiology offices (35% of all providers), on the other hand, have the lowest excess charge ratio at the procedure level (2.59). Most notably, these providers undercharge at the practice level, a trait that is not observed in any other provider segment in our analysis. The average payment is almost $100 more for cluster #1, cardiology offices ($364) compared to cluster #4, cardiology offices ($265). This latter group of providers serves a significantly higher number of beneficiaries. The average service volume and number of unique beneficiaries are 324 and 279, respectively (compared to 108 and 87 for cluster #1). Geography wise, there are more rural offices in the location mix for cluster #4 and a higher % of dual-eligible beneficiaries. In a nutshell, cluster #4 providers, although small in number, are serving a larger patient base in more rural areas and are billing Medicare more. A scatter plot of selected attributes for clusters #1 and #4 are illustrated in Fig. 2.

### HCPCS GO202: screening mammography, producing a direct digital image, bilateral, all views

Results of the two-step cluster analysis are provided in Table 5, highlighting the variables with significant differences among clusters. The cluster silhouette measure of cohesion and separation is 0.4 (considered fair).

**Table 5:** Healthcare Provider Clusters: HCPCS Code G0202

		Cluster Centroids (Average)			
	Cluster # Relative Size (Frequency)	1 12.1% (2171)	2 27.7% (4834)	3 3.5% (620)	4 56.4% (9848)
**Healthcare Provider Attributes**	Provider Type	Diagnostic Radiology (76.9%) Obstetrics/Gynecology (20.64%)	Diagnostic Radiology (66.1%) Family Practice (15.76%) Internal Medicine (12.83%)	Diagnostic Radiology (43.9%) Independent Diagnostic Testing Facility (40.81%)	Diagnostic Radiology (97.9%)
**Medical Procedure Attributes**	Place of Service	Office (55%)	Office (100%)	Office (96.5%)	Office (91.2%)
	Service Volume	666.22	178.07	602.31	263.33
	Unique Beneficiary	664.40	177.61	534.18	263.30
	Service/Unique Beneficiary	1.00	1.00	1.29	1.00
	Medicare Allowed Amount	$81.13	$125.99	$101.08	$35.32
	Medicare Payment Amount	$79.22	$123.12	$98.88	$34.57
	Excess Charge Ratio	2.65	2.30	2.43	3.04
**Medical Practice Attributes**	Total Unique HCPCS codes submitted	64.80	147.08	149.50	171.38
	Total Unique Beneficiary	1574.76	1903.67	3011.00	2931.56
	Overall Excess Charge Ratio	3.00	3.28	4.03	3.96
**Demographic and Socio-economic Attributes**	Beneficiary Average Risk Score	1.02	1.37	1.26	1.59
	Beneficiary Dual Percentage	16.95	23.31	20.64	27.82
	Location Mix (Urban: Rural: Very Rural)	(94.5%: 5.3%: 0.2%)	(87.4%: 10.3%: 2.3%)	(87.9%: 11.5%: 0.6%)	(83.9%: 14.0%: 2.2%)

In contrast to the previous two medical procedures, the segmentation for this procedure is based on a combination of patient and provider characteristics. Healthcare providers that fall into cluster #1 generally service the lowest risk (average beneficiary risk score of 1.02) and dual-eligible (16.95%) patients. Mammograms constitute a sizeable portion of these providers’ services (on average, 666 out of 1575 unique beneficiaries). Located mostly in urban areas, these providers have the lowest excess charge ratio at the practice level.

Healthcare providers in cluster #2 have the lowest procedure level excess charge ratio (2.30) and the second lowest (3.28) excess charge ratio at the practice level. Only a small % of their patients (on average, 177 out of 1903 unique beneficiaries) receive the service. This cluster receives the highest average payment ($123). Cluster #3 represents a very small number (only 3.5%) of providers. Even though the excess charge ratio is the highest at the practice level (4.03) for these providers, it is not as much for the procedure (2.43). These providers get paid $98.88 for their service. On average, they also provide more than one service/beneficiary.

Cluster #4, consisting predominantly of diagnostic radiology offices, is the largest healthcare provider group, representing more than half (56.4%) of providers of screening mammography. These providers serve the highest risk (1.59) and dual-eligible (27.82%) patients. Medicare pays these providers significantly less than their counterparts ($34.57).

**Fig. 3:**
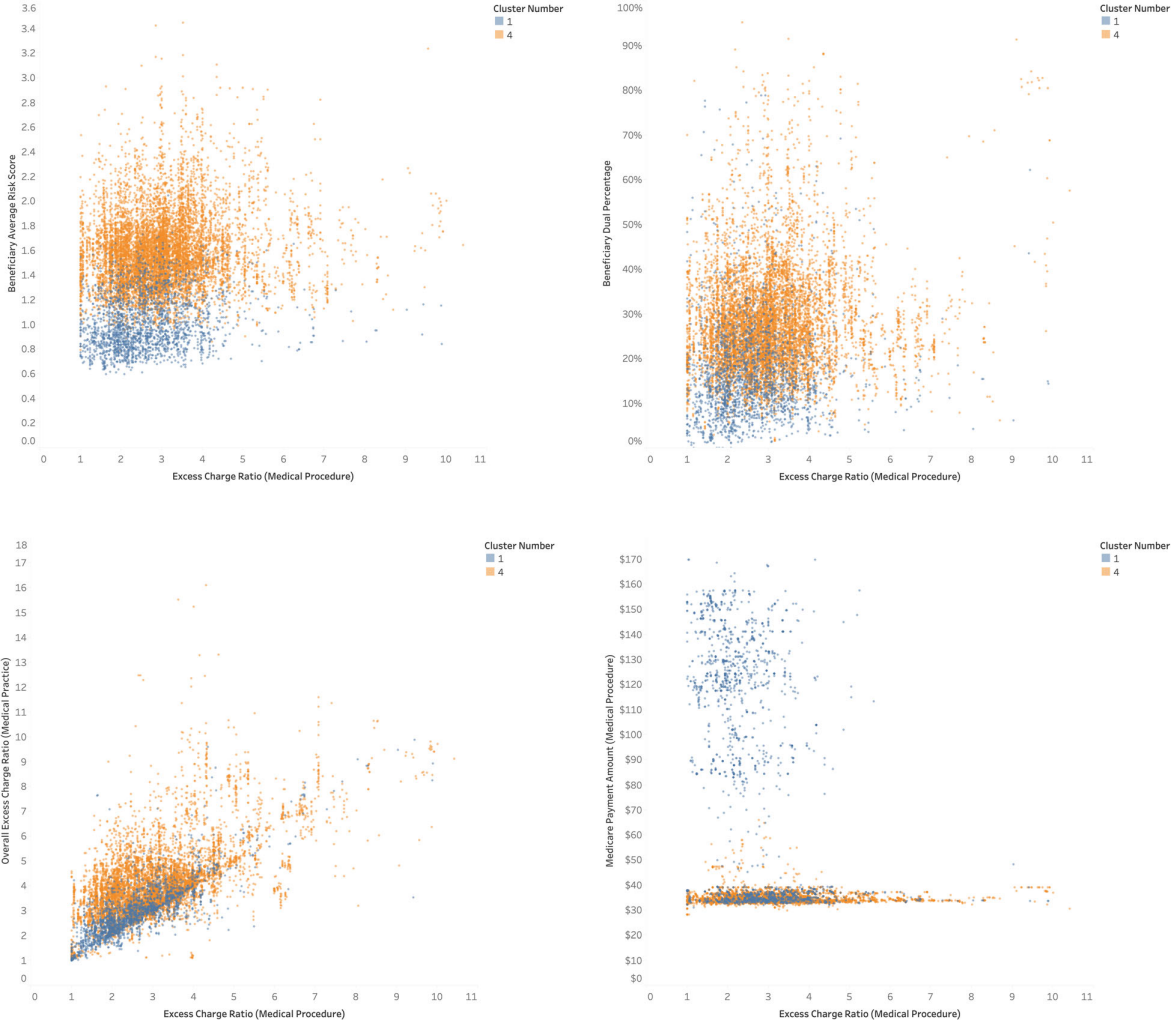
Cluster Differences for Screening Mammograms (Medical Procedure 66,984)

In general, providers with a wider base of other services (clusters #2 and #3) seem to have the least amount of excess charge for the procedure. It is not apparent why these two clusters are paid much better ($123 and $99, respectively) than their peers. Cluster #4 deals with a poorer, riskier patient base in less urban areas. Providers in this cluster tend to have the highest excess charge and are paid considerably less compared to their peers. A scatter plot of selected attributes for clusters #1 and #4, which have significant differences in their attributes, is illustrated in Fig. 3.

### Validation

For each of the three medical procedure codes, we created three random subsamples, taking 15, 30, and 45% of the entire data set. These are compared with the results obtained using the main data set. Details of the validation results are provided in the Appendix. For procedure codes 66,984 and 78,452, the number of clusters is the same as that obtained using the entire data set, and the cluster centroids do not exhibit any difference from one another or from the clusters obtained from the entire data set. The cluster centroids are consistent among the subsamples. For procedure code G0202, all three smaller subsamples yielded one less cluster compared to the one using the entire dataset. A closer inspection reveals that the two larger clusters across the main data set and the subsamples are similar, whereas the two smaller clusters from the main data correspond to one cluster in the subsamples. In other words, the difference between these two smaller clusters becomes significant only in a larger data set.

## Discussion

**Fig. 4:**
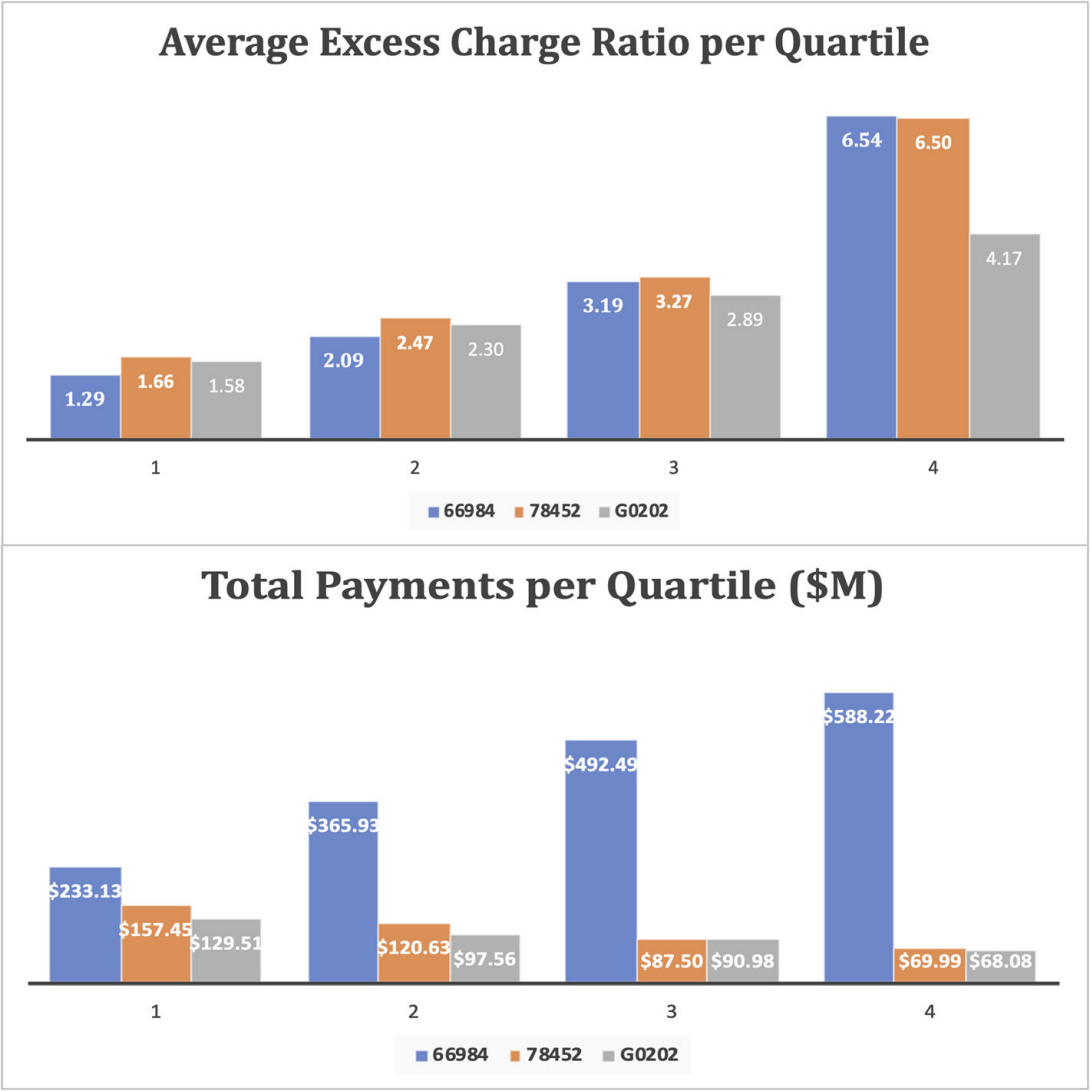
Excess Charge Ratio and Total Payment Per Excess Charge Quartiles

For the three medical services discussed in this article, when healthcare providers are ranked into four quartiles in terms of the procedure level excess charge ratio, providers in the topmost quartile charge 4 to 6.5 times the Medicare allowable limit (Fig. 4). This is significantly higher than the lower 3 quartiles, where the excess charge ratio ranges from 1.29 to 3.27. Of these procedures, cataract surgery (HCPCS 66984) and screening mammograms (HCPCS G0202) can be considered quite commonplace for Medicare beneficiaries. Provider excess charges for such services require further exploration. Privately insured, out-of-network, and uninsured patients do not enjoy the benefits of bargaining by the big networks and are often billed the full amount. Without a voluntary discount from the providers, such high medical bills are often the cause of significant financial distress for uninsured and out-of-network patients. This is especially important in the case of cataract surgery, where the highest excess charge quartile of providers are getting reimbursed almost the same as quartiles 1 and 2 taken together. Given that only about 25% of providers are billing significantly higher prices compared to their peers, a naturally occurring question involves the particular factors underlying such differences, and more importantly, if there are any inefficiencies in the system.

In our search for such inefficiencies, provider gender, affiliation (whether affiliated with an organization or operating as an individual), as well as the racial/ethnic makeover of the patient base did not emerge as important differentiating variables. This is definitely a positive discovery, as it indicates that inequities are mostly attributed to practice-specific and patient socioeconomic characteristics. Our segmentation of the provider base highlights factors with important policy-level implications.

### Site-based payment differential

A key finding from our study illustrates the role of site-based differential payments in terms of creating inefficiencies in healthcare delivery. These inefficiencies are manifest differently in cataract surgery and nuclear imaging. Nonetheless, they underscore the need to revise payment policies.

#### Cataract surgery: office-based vs ASC

HCPCS code 66984 was the most prevalent form of cataract surgery performed for Medicare fee-for-service beneficiaries. In 2014, 6.89 billion such services related to non-complex cataract surgeries were performed. From 2001 to 2014, the volume of cataract surgery has undergone a large shift from hospital-operated outpatient departments (HOPD) to ASCs [49]. The primary reason for such a shift involves greater patient convenience, physician productivity, lower out-of-pocket costs for patients and reduced costs for insurers. In a March 2018 report to Congress, MedPAC (Medicare Payment Advisory Commission) reported that a payment rate of $992 (in contrast to a payment rate of $1912) to HOPDs has been beneficial [50]. More recently, office-based surgeries (i.e., performed at the ophthalmologist’s office, which is equipped to perform certain surgical procedures) have been proved to be safe, effective, and less expensive [51]. In 2015, CMS solicited input from stakeholders regarding the payment structure for office-based cataract surgeries. In their response, in addition to safety and security concerns about patients, physician associations have voiced concerns about investments in equipment, technology, anesthesia, and nursing staff [52]. Also, due to the higher volume of surgeries in ASCs, the economies of scale are argued to be higher in ASCs and are thus more efficient for providers.

**Fig. 5:**
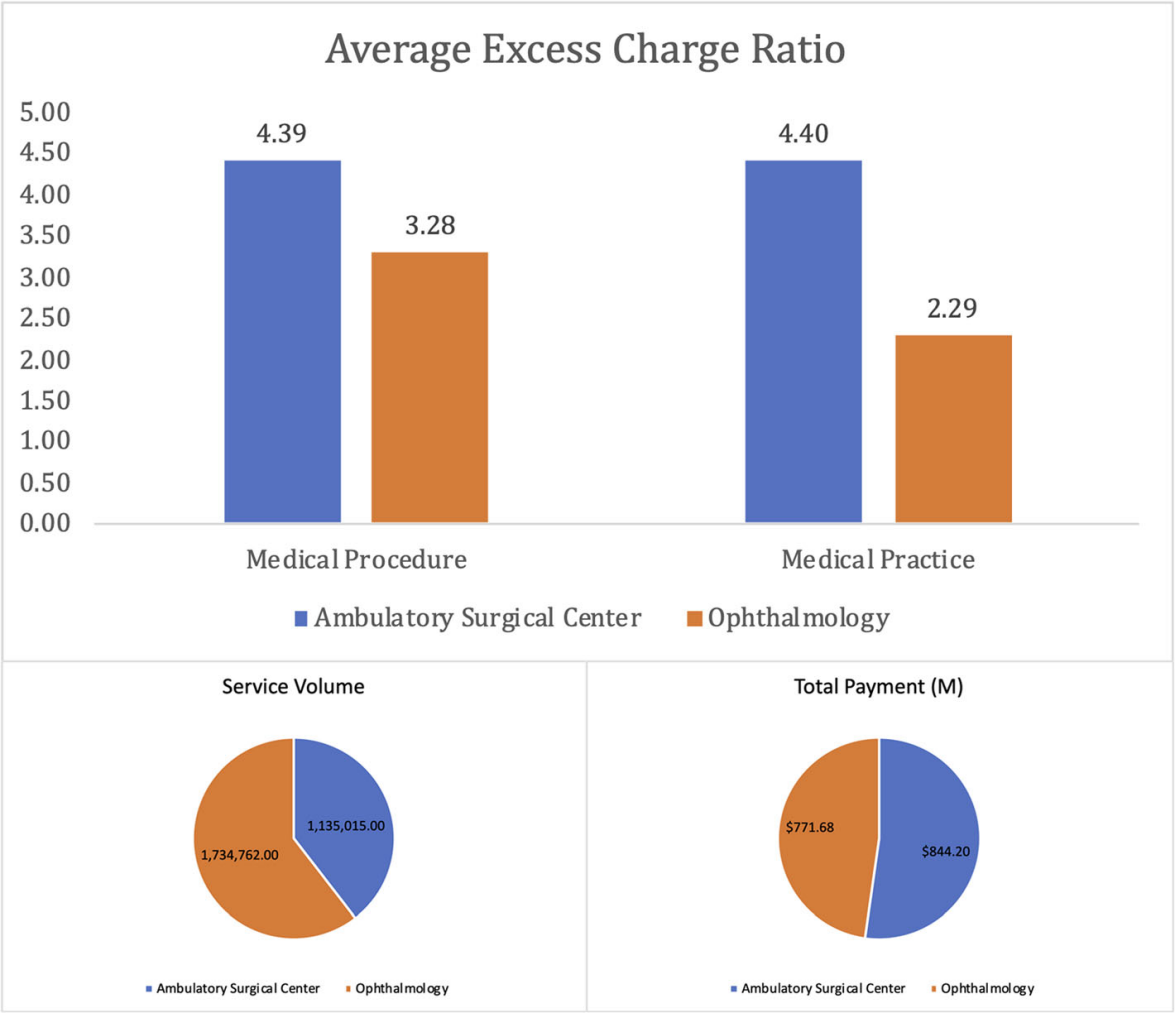
ASCs (*N* = 1708) vs Ophthalmologists (*N* = 8896)

In 2014, almost 1.05 M more office-based cataract surgeries were performed (compared to ASCs) for Medicare beneficiaries at a $69 M lower price tag (Fig. 5). This is in contrast with a recent study [49] in a privately managed care network, where 73% of the cataract surgeries were performed in ASCs in the same year. With an average volume of 623 compared to 223 for office-based cataract surgery, ASCs certainly benefit from better economies of scale. Despite this, ASCs not only have a higher degree of excess charge at both the procedure and practice levels (compared to ophthalmologists), but also get reimbursed at higher amounts. The juxtaposition of private and public insurance in utilizing ASC- vs office-based non-complex cataract surgeries poses a significant question. Do office-based surgeries offer a viable and cost-effective option for all insurance providers without any compromise to quality? This can only be answered by analyzing private insurance claims and payment data in conjunction with public data.

#### Nuclear cardiac imaging: office vs facility

HCPCS 78452 is a bundled service that includes myocardial perfusion imaging and multiple studies at rest and/or stress (exercise or pharmacologic) and/or redistribution and/or rest reinjection. Even though the bundling of procedures in 2010 (to include stress tests) has resulted in payment reductions since 2013 [50], there is still a huge payment differential among facility and office-based services. Procedures performed at cardiologist offices were reimbursed at an average service price of $347 compared to $60 for cardiology facilities or diagnostic radiology centers. In 2014, for almost the same volume of services (983,788 in offices vs 911,044 in facilities), such a site-based differential resulted in excess payments of $285.72 M for procedures performed at offices.

**Fig. 6:**
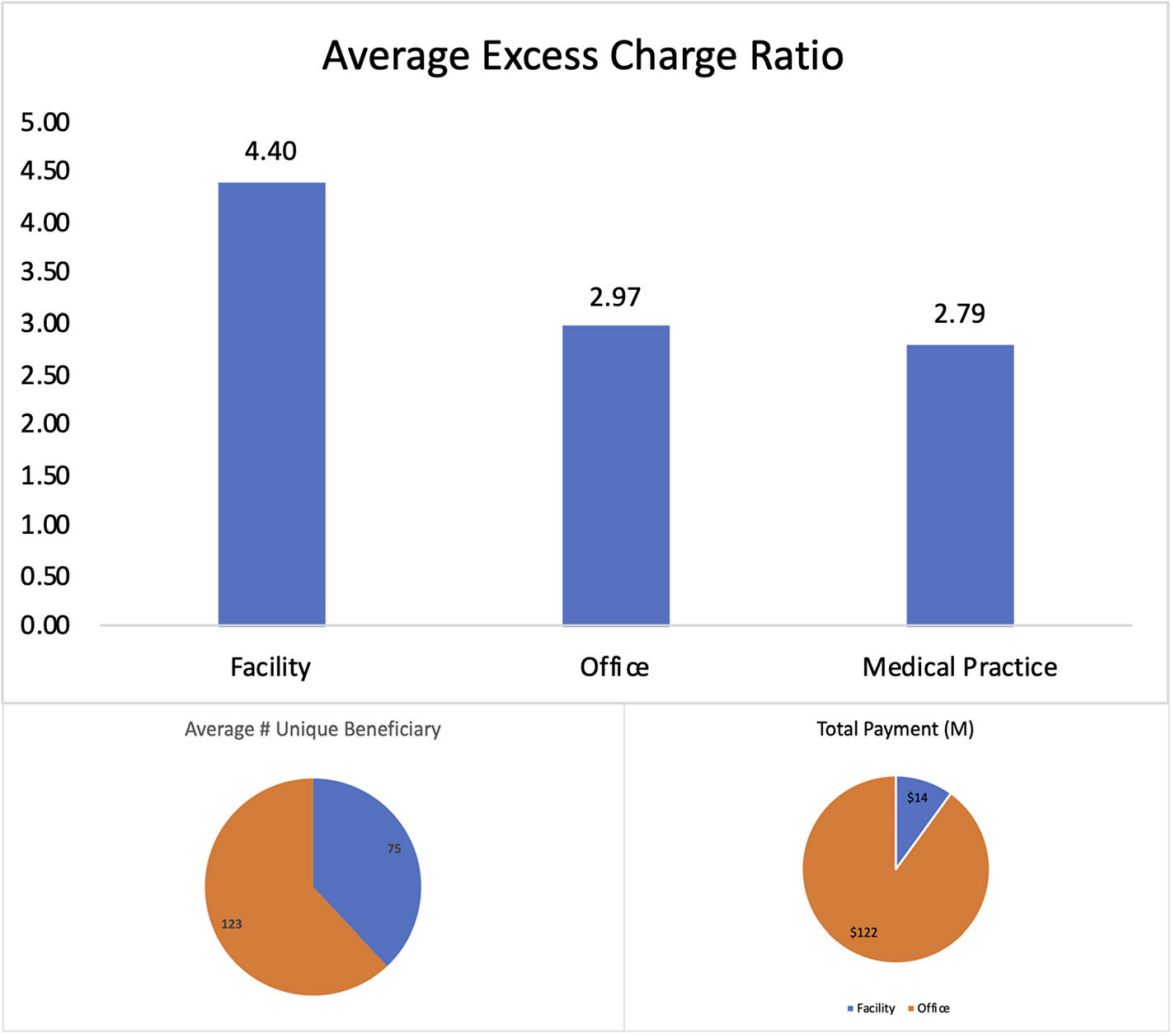
Nuclear Cardiac Imaging: Site-Based Differences in Volume, Total Payment, and Excess charge Ratio for Providers with Both Office and Facility Based Claims (*N* = 2997)

Observably this results in a shift to more office visits. In 2014, 2997 providers billed for both facility and office-based services. On average, the same provider billed for 123 unique patients in the office versus billing for 75 in a facility (Fig. 6). These office-based claims resulted in $108 M more in payments to these providers. Interestingly, the excess charge ratio for facility-based claims is higher than that for office-based claims, with the latter being almost the same as a practice’s overall excess charge. Taken together, the statistics may be indicative of the potential for cost-savings from site-neutral payment policies. On the other hand, it is also possible that the higher excess charge for facility-based services is a sign that the Medicare reimbursement policy is not adequate to cover the costs.

### Screening mammograms: are variations related to patient demographics?

**Fig. 7:**
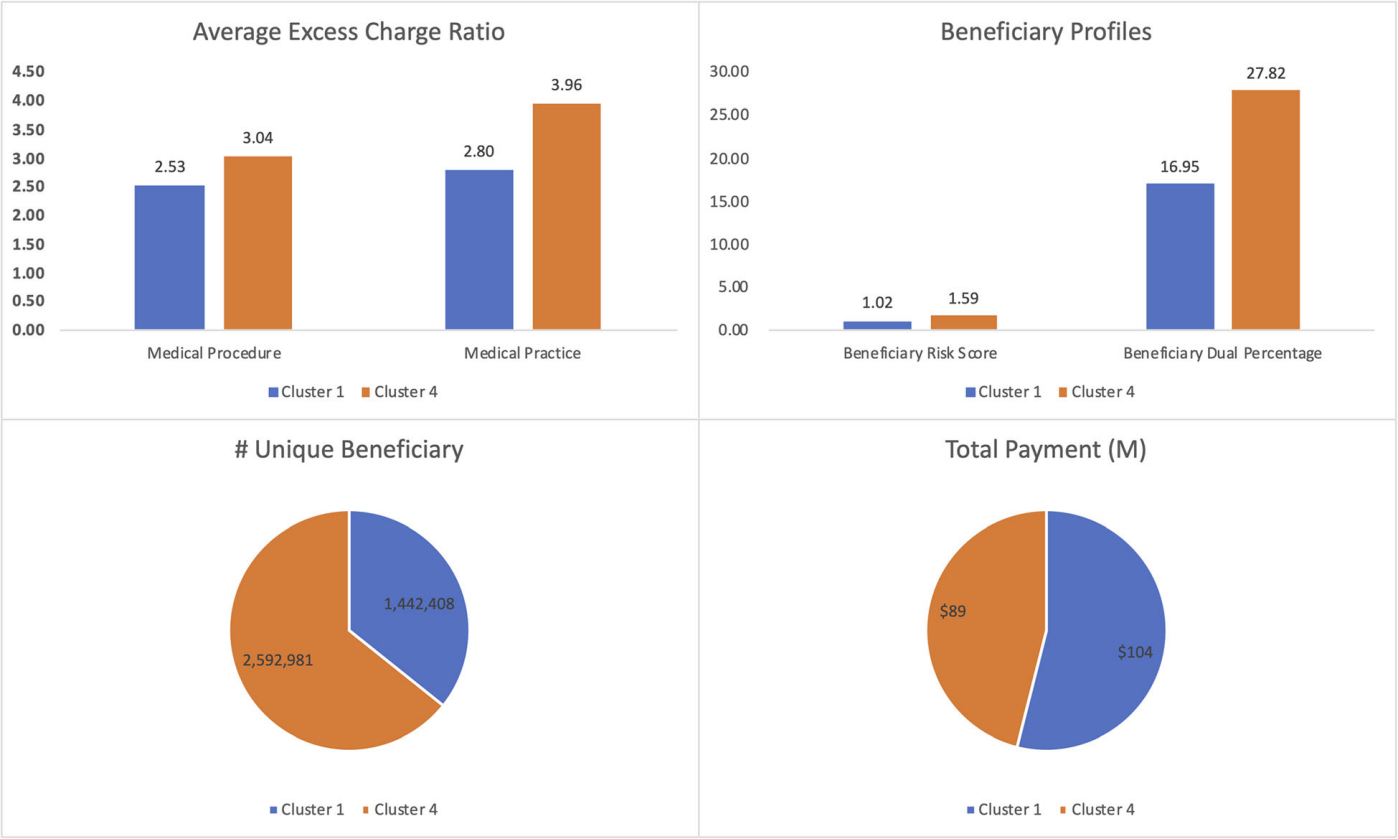
Differences Between Two Largest Provider Segments (Cluster 1, *N* = 2171, Cluster 4, *N* = 9848) of Screening Mammograms

HCPCS G0202, i.e., screening mammograms, falls under preventive health benefits. This is a standard procedure that (at least theoretically) should not demonstrate wide payment variation. However, as evidenced by the analysis, a considerable amount of differential payment exists among different provider segments. The most alarming of them being between the two largest healthcare provider segments, provider cluster #1 (2171 providers) and provider cluster #4 (9848 providers). As seen in Fig. 7, the latter segment’s patient base has a higher-than-average risk score and dual eligibility. In spite of serving almost 1 M more unique beneficiaries, the share of payments for providers in cluster #4 is $25 M less. Subsequently, their excess charge amount is also higher. Once again, we observe that this segment has more excess charges at the both procedure and practice levels, which may be indicative of inadequate reimbursement by CMS**.**

## Conclusion

In this study, we conducted a novel application of two-step cluster analysis utilizing multiple healthcare data sources to uncover provider behavior patterns with respect to overbilling in three prominent medical procedures covered by US Medicare. Before we conclude, we would like to acknowledge the limitations of our study. Due to privacy concerns, data from providers with a very small base of beneficiaries are suppressed by CMS and are subsequently excluded from the cluster analysis. The provider payment and utilization information is based on data from 2014. A follow-up analysis of data from the following years seems to hold with respect to our main findings. It is possible that incremental changes in behavior patterns have taken place, which can only be confirmed by a longitudinal study. Also, we have used a combination of urban/rural/very rural categorization using zip codes by CMS and the dual-eligible beneficiary percentage as a proxy for the socioeconomic status of a provider’s patient base. Based on our understanding of the effect of key attributes on excess charge, our next step is to use statistical and machine learning methods in predicting healthcare providers’ excess charge behavior.

In this research, we asked the following question: what does excess charge inform us about provider billing patterns for medical procedures with high payment variation? Our results highlight that for each of the three medical procedures chosen for this analysis, there are segments of providers who, despite serving a large pool of beneficiaries, receive comparatively less Medicare reimbursements per service. These providers also have higher excess charge ratios. Since federal legislation protects Medicare beneficiaries from balance billing – i.e., charging the patient directly for the difference between the allowed and charge amounts – these excess charges do not result in collection from patients. However, a plausible hypothesis for such billing behavior is that “these charges generally reflect what providers bill to other patients—privately insured, self-pay, out-of-network, and uninsured” [3]. Earlier research has supported this claim. Private insurance data are not always available. However, the findings from our study, when coupled with another research study that had access to private insurance data [49], seems to support this notion as well. Our analysis finds ambulatory surgery centers (ASCs) to have a very high excess charge ratio for cataract surgery. ASCs serve a smaller share of Medicare patients for cataract surgery compared to Ophthalmology offices. However, this is quite in contrast with private insurance where a much higher proportion of cataract surgeries are performed in ASCs [49], implying possible cost-shifting [13, 14] to private insurance.

Furthermore, the practice of excess charge over the years has ultimately led to higher payments for private insurance without any significant improvement in care quality or efficiency [53]. This has also meant higher prices for consumers of health services. In this regard, Medicare fee schedule has a very significant effect in setting private commercial markets, as physician payments are shown to be tied to Medicare pricing in almost 75% of the services provided [54]. Average commercial prices for physicians’ services are substantially higher than Medicare Fee-for-Service (FFS). This difference is even higher for specialty services [32]. Research has shown that physician payment rates are higher when the provider competition is less and vice versa. Also, physicians charging higher prices definitely have higher market power [17]. While they may provide better care coordination and management, they do not fare better in terms of overall healthcare quality or efficiency [22]. As shown in our study, excess charge for basic preventive services such as screening mammograms are higher in rural areas, where presumably private insurance companies have less market power because of lack of competition. This would imply that private or uninsured patients would be charged higher prices for this service. The ultimate result may be that these patients do not avail this preventive service and may face subsequent adverse health outcomes. In other words, excess charge may indirectly lead to urban-rural healthcare disparities observed in other studies [55]. In spite of federal and state governments’ recent efforts to protect private (non-Medicare) healthcare consumers from surprise bills [56], only a handful of US states provide comprehensive protection as of 2019 [57].

In summary, our findings suggest that excess charge is essentially a signal of a provider’s bargaining power in the private insurance market. The immediate effect of such overbilling is observable in financial distress for uninsured and underinsured patients [58, 59]. The long-term effect of excess charge seems to be an increase in price. There have been calls for price transparency [17], and more importantly, for having an upper limit to excess charge [53] or capping payment at 125% of Medicare rates [60]. Recent research suggests that the market power resulting from such efforts leads to reduced prices for standard medical procedures [61]. In this respect, our research underscores the need for price transparency in both public and private insurance markets and reinforces the call for limited excess charge.

## Supplementary Information

**Additional file 1:** **Table S1.** Healthcare Provider Cluster Validation: HCPCS Code 66984 (3 random subsamples). **Table S2.** Healthcare Provider Cluster Validation: HCPCS Code 78452 (3 random subsamples). **Table S3.** Healthcare Provider Cluster Validation: HCPCS Code G0202 (3 random subsamples).

## Data Availability

All data used in this research are publicly available at https://www.cms.gov/Research-Statistics-Data-and-Systems/Statistics-Trends-and-Reports/Medicare-Provider-Charge-Data/Physician-and-Other-Supplier

## Abbreviations

BIC: Bayesian information criterion (BIC)
CMS: Center for medicare and medicaid services
HCPCS: Healthcare common procedure coding systems
HOPD: Hospital operated outpatient departments
ASC: Ambulatory surgical center
