# Supplementary material for: Discovering healthcare provider behavior patterns through the lens of Medicare excess charge
Source: BMC Health Serv Res. 2021 Jan 4;21:2. doi: 10.1186/s12913-020-05876-1 (PMC7780410; doi:10.1186/s12913-020-05876-1)
Supplement: Supplementary file 1 — Additional file 1: Table S1. Healthcare Provider Cluster Validation: HCPCS Code 66984 (3 random subsamples). Table S2. Healthcare Provider Cluster Validation: HCPCS Code 78452 (3 random subsamples). Table S3. Healthcare Provider Cluster Validation: HCPCS Code G0202 (3 random subsamples). [file 12913_2020_5876_MOESM1_ESM.pdf]

Table A.1: Healthcare Provider Cluster Validation: HCPCS Code 66984 (3 random subsamples)

			Cluster Centroids (Average)		
**Cluster #: Relative Size**		Sub sample 1	1: 31%	2: 11%	3: 58%
		Sub sample 2	1: 25.2%	2:10.6%	3: 64%
		Sub Sample 3	1: 26.30%	2: 11.90%	3: 61.80%
**Healthcare Provider Attributes**	Provider Type	Sub sample 1	Optometry (86.1%)	Ambulatory Surgical Center (99.6%)	Ophthalmology (99.9%)
		Sub sample 2	Optometry (86.1%)	Ambulatory Surgical Center (99.6%)	Ophthalmology (99.9%)
		Sub Sample 3	Optometry (86.5%)	Ambulatory Surgical Center (100%)	Ophthalmology (99.9%)
**Medical Procedure Attributes**	Place of Service	Sub sample 1	Office (94.9%)	Facility (99.9%)	Facility (97.9%)
		Sub sample 2	Office (94.9%)	Facility (99.9%)	Facility (97.9%)
		Sub Sample 3	Office (95.1%)	Facility (100%)	Facility (98.2%)
	Service Volume	Sub sample 1	758.5	631.1	188.5
		Sub sample 2	758.48	631.12	188.47
		Sub Sample 3	666.94	667.34	192.64
	Unique Beneficiary	Sub sample 1	32.3	412.2	111.4
		Sub sample 2	32.67	430.90	112.65
		Sub Sample 3	34.12	434.79	115.96
	Service/Unique Beneficiary	Sub sample 1	27.3	1.5	1.6
		Sub sample 2	25.02	1.51	1.59
		Sub Sample 3	23.31	1.52	1.57
	Medicare Allowed Amount	Sub sample 1	$85.9	$965.5	$643.3
		Sub sample 2	$88.06	$969.89	$643.84
		Sub Sample 3	$91.21	$965.25	$643.18
	Medicare Payment Amount	Sub sample 1	$67.0	$748.2	$498.0
		Sub sample 2	$68.59	$751.29	$498.27
		Sub Sample 3	$71.04	$748.29	$497.78
	Excess Charge Ratio	Sub sample 1	3.5	4.5	3.3
		Sub sample 2	3.75	4.35	3.27
		Sub Sample 3	3.25	4.41	3.27
**Medical Practice Attributes**	Total Unique HCPCS codes submitted	Sub sample 1	21.4	97.6	39.2
		Sub sample 2	21.59	102.17	39.66
		Sub Sample 3	21.59	98.44	39.49
	Total Unique Beneficiary	Sub sample 1	531.6	983.6	961.2
		Sub sample 2	520.96	1071.84	963.38
		Sub Sample 3	527.04	1,041.69	976.92
	Overall Excess Charge	Sub sample 1	1.4	4.6	2.3
		Sub sample 2	1.44	4.41	2.29
		Sub Sample 3	1.43	4.37	2.30
Demographic and Socio-Economic attributes	Location Mix (Urban: Rural: Very Rural)	Sub sample 1	(50%: 39%: 11%)	(88%: 11%: 1%)	(88%: 10%: 2%)
		Sub sample 2	(50%, 39%,11%)	(88%, 11%,1%)	(89%, 10%, 2%)
		Sub Sample 3	(52%: 38%: 10%)	(87%,11%,2%)	(87%,111%,2%)
	Beneficiary Dual Percentage	Sub sample 1	18.6	15.5	16.3
		Sub sample 2	18.28	14.61	16.27
		Sub Sample 3	18.33	14.80	16.46

Subsamples 1, 2, and 3 represent 15%, 30%, and 45% of the dataset pertaining to HCPCS code 66984. The number of clusters is the same as presented in the main paper. The cluster centroids do not exhibit any difference from one another and from the clusters obtained from the entire data set (presented in the main paper).

Table A.2: Healthcare Provider Cluster Validation: HCPCS Code 78452 (3 random subsamples)

Cluster # Relative Size			Cluster Centroids (Average)			
		Sub sample 1	1: 34.2%	2:37.5%	3:20.1%	4: 8.1%
		Sub sample 2	1: 37.9%	2: 37.8%	3: 20.3%	4: 4.1%
		Sub Sample 3	1: 37%	2: 38.8%	3: 19.5%	4: 4.7%
**Healthcare Provider Attributes**	Provider Type	Sub sample 1	Cardiology (89.8%)	Cardiology (90%)	Diagnostic Radiology (97%)	Cardiology (54.7%)
		Sub sample 2	Cardiology (84%)	Cardiology (90.3%)	Diagnostic Radiology (97.5%)	Cardiology (70%)
		Sub Sample 3	Cardiology (83.9%)	Cardiology (90.2%)	Diagnostic Radiology (97.6%)	Cardiology (68.4%)
**Medical Procedure Attributes**	Place of Service	Sub sample 1	Office (100%)	Facility (98.5%)	Facility (98.2%)	Office (92.2%)
		Sub sample 2	Office (100%)	Facility (97.7%)	Facility (97.5%)	Office (78.8%)
		Sub Sample 3	Office (100%)	Facility (94.4%)	Facility (97.7%)	Office (75.3%)
	Service Volume	Sub sample 1	110.0	96.2	56.4	241.5
		Sub sample 2	117.4	94.3	55.6	320.0
		Sub Sample 3	110.0	93.4	53.5	348.8
	Unique Beneficiary	Sub sample 1	86.703	84.846	51.169	279.283
		Sub sample 2	109.2	95.6	56.1	227.3
		Sub Sample 3	109.2	92.9	53.3	318.4
	Service/Unique Beneficiary	Sub sample 1	1.0	1.0	1.0	1.1
		Sub sample 2	1.0	1.0	1.0	1.2
		Sub Sample 3	1.0	1.0	1.0	1.2
	Medicare Allowed Amount	Sub sample 1	$457.8	$79.8	$81.2	$384.5
		Sub sample 2	$457.5	$79.8	$82.5	$314.7
		Sub Sample 3	$474.5	$80.3	$81.9	$311.2
	Medicare Payment Amount	Sub sample 1	$352.6	$60.1	$60.4	$295.7
		Sub sample 2	$352.4	$60.1	$61.6	$241.8
		Sub Sample 3	$365.6	$60.5	$61.1	$239.3
	Excess Charge Ratio	Sub sample 1	2.6	4.1	3.7	3.2
		Sub sample 2	2.6	4.0	3.8	4.8
		Sub Sample 3	2.6	4.1	3.9	3.8
**Medical Practice Attributes**	Total Unique HCPCS codes submitted	Sub sample 1	67.0	61.4	179.6	114.9
		Sub sample 2	72.9	61.3	180.1	108.3
		Sub Sample 3	72.7	61.2	181.2	102.0
	Overall Excess Charge	Sub sample 1	2.5	2.8	3.9	2.9
		Sub sample 2	2.5	2.8	3.9	3.7
		Sub Sample 3	2.5	2.9	4.0	3.6
Demographic and Socio-Economic attributes	Beneficiary Dual Percentage	Sub sample 1	21.1	23.1	29.4	24.4
		Sub sample 2	21.8	23.4	28.6	27.2
		Sub Sample 3	22.7	22.9	28.8	25.2
	Location Mix (Urban: Rural: Very Rural)	Sub sample 1	(94%: 6%: 0%)	(87%: 12%: 1%)	(85%: 14%: 1%)	(84%: 16%: 0%)
		Sub sample 2	(92%: 8%: 0%)	(87%: 11%: 2%)	(82%: 15%: 3%)	(82%: 17%: 1%)
		Sub Sample 3	(91%: 8%: 1%)	(87%: 12%: 1%)	(82%: 15%: 3%)	(80%: 19%: 1%)

Subsamples 1, 2, and 3 represent 15%, 30%, and 45% of the dataset pertaining to HCPCS code 78452. The number of clusters is the same as presented in the main paper. The cluster centroids do not exhibit any difference from one another and from the clusters obtained from the entire data set (presented in the main paper).

Table A.3: Healthcare Provider Cluster Validation: HCPCS Code G0202 (3 random subsamples*)*

Cluster # Relative Size			Cluster Centroids (Average)		
		Sub sample 1	1: 11%	2: 31%	3: 58%
		Sub sample 2	1:5%	2: 34%	3: 62%
		Sub Sample 3	1: 7%	2: 35%	3: 65%
**Healthcare Provider Attributes**	Diagnostic Radiology: Obstetrics/Gynecology: Family Practice: Internal Medicine	Sub sample 1	(96%, <1%: 1%: 2%)	(64%: 10%, 14%,12%)	(100%: 0%: 0%: 0%)
		Sub sample 2	(87%, 4%, 4%, 5%)	(67%: 8%: 14%: 11%)	(99.8% :<1%: <1%: <1%)
		Sub Sample 3	(91%: 4%: 3%: 2%)	(66%: 8%: 15%: 11%)	(99.9%: <1%: <1%: <1%)
**Medical Procedure Attributes**	Place of Service	Sub sample 1	Office (58.7%)	Office (100%)	Facility (91.5%)
		Sub sample 2	Office (60%)	Office (99%)	Facility (92%)
		Sub Sample 3	Office (62%)	Office (97%)	Facility (95%)
	Service Volume	Sub sample 1	868.29	160.53	269.75
		Sub sample 2	819.01	220.64	305.71
		Sub Sample 3	923.64	185.63	299.60
	Unique Beneficiary	Sub sample 1	841.87	160.21	269.73
		Sub sample 2	755.33	219.65	305.68
		Sub Sample 3	871.08	184.86	299.59
	Service/Unique Beneficiary	Sub sample 1	1.10	1.00	1.00
		Sub sample 2	1.26	1.00	1.00
		Sub Sample 3	1.21	1.00	1.00
	Medicare Allowed Amount	Sub sample 1	$81.04	$124.48	$35.32
		Sub sample 2	$97.99	$124.80	$35.40
		Sub Sample 3	$99.45	$124.62	$35.36
	Medicare Payment Amount	Sub sample 1	$79.30	$121.63	$34.57
		Sub sample 2	$95.85	$121.91	$34.65
		Sub Sample 3	$97.28	$121.73	$34.61
	Excess Charge Ratio	Sub sample 1	2.69	2.30	3.05
		Sub sample 2	2.44	2.30	3.07
		Sub Sample 3	2.49	2.30	3.03
**Medical Practice Attributes**	Total Unique HCPCS codes submitted	Sub sample 1	107.19	130.59	168.85
		Sub sample 2	143.40	129.79	162.82
		Sub Sample 3	132.67	130.14	164.82
	Total Unique Beneficiary	Sub sample 1	2,632.94	1,647.81	2,907.56
		Sub sample 2	3,294.29	1,718.53	2,859.03
		Sub Sample 3	3,076.44	1,674.54	2,874.20
	Overall Excess Charge	Sub sample 1	3.67	3.17	3.94
		Sub sample 2	3.88	3.16	3.95
		Sub Sample 3	3.71	3.20	3.93
Demographic and Socio-Economic attributes	Beneficiary Average Risk Score	Sub sample 1	1.15	1.30	1.57
		Sub sample 2	1.24	1.28	1.55
		Sub Sample 3	1.19	1.29	1.55
	Beneficiary Dual Percentage	Sub sample 1	17.29	22.07	27.43
		Sub sample 2	20.25	21.88	26.86
		Sub Sample 3	19.99	21.88	27.06
	Location Mix (Urban: Rural: Very Rural)	Sub sample 1	(95%: 5%: <1%)	(87%: 11%: 2%)	(84%: 14%: 2%)
		Sub sample 2	(90%: 10%, <1%)	(89%, 9%, 2%)	(85%, 12%, 2%)
		Sub Sample 3	(92%: 8%, <1%)	(89%, 9%, 2%)	(84%, 13%, 3%)

Subsamples 1, 2, and 3 represent 15%, 30%, and 45% of the dataset pertaining to HCPCS code G0202. The number of clusters is 3 (compared to 4 in the main paper), the cluster centroids are consistent among the subsamples. The larger 2 clusters (Clusters #2, and #3 in the subsamples) are the same as clusters #2 and #4 in the main paper. The smallest cluster (#1 in the subsample) seems to correspond to clusters #1 and #3 in the main paper. In other words, the smallest of the cluster show sufficient differences within its members only in a large dataset and subsequently divides into 2 clusters.
